# Longitudinal Association Between Depressive Symptoms and Cognitive Function Among Older Adults: A Latent Growth Curve Modeling Approach

**DOI:** 10.3389/ijph.2022.1605124

**Published:** 2022-09-23

**Authors:** Zihan Gao, Cuiping Liu, Li Yang, Xinyi Mei, Xiao Wei, Jinke Kuang, Kexin Zhou, Mengfan Xu

**Affiliations:** ^1^ School of Nursing, Qingdao University, Qingdao, China; ^2^ School of Nursing, Wuhan University of Science and Technology, Wuhan, China

**Keywords:** longitudinal study, cognitive function, depressive symptoms, latent growth curve model, old adults

## Abstract

**Objectives:** Although the evidence from numerous longitudinal studies has indicated a remarkable change in cognitive function (CF) and depressive symptoms (DS) over time, the parallel latent growth curve model (LGCM) has seldom been used to simultaneously investigate the relationship between their change trajectories. This study aimed to examine whether a change in DS was associated with CF over time using an LGCM.

**Methods:** Data were collected from the Chinese Longitudinal Healthy Longevity Survey’s 2011, 2014, and 2018 waves. A parallel LGCM examined the association between CF and DS.

**Results:** The multivariate conditioned model’s goodness of fit supported the validity of the longitudinal model (Tucker-Lewis index [TLI] = 0.90, comparative fit index [CFI] = 0.96, root mean square error of approximation [RMSEA] = 0.04). The results showed that the CF intercept was positively to the DS slope (*β* = 0.42, *p* = 0.004). The CF and DS slopes were significantly linked (*β* = −0.65, *p* = 0.002).

**Conclusion:** The findings expand the knowledge about CF’s effect on DS in older adults.

## Introduction

Depressive symptoms (DS) and cognitive function (CF) impairment are globally recognized as the most common mental disorders in older adults [1, 2]. A chronic unhealthy mental state caused by depression or lack of interest in external stimuli causes significant disturbance to older adults’ lives and leads to adverse health outcomes, such as mortality, chronic disease incidence rate, economic burden, functional disability, and decline in quality of life (QOL) [1,3,4]. The prevention and treatment of depressive symptoms are crucial for older adults [5]. Cognitive impairment is often manifested as the impairment of one or more abilities related to perception, attention, memory, and thinking [6]. Over 46 million older adults suffer from cognitive impairment, which imposes a heavy burden on families and society [7,8]. Moreover, the cumulative dementia incidence rate is 14.9% in older adults with mild cognitive impairment aged above 65 years [9]. Exploring the different development trajectories and influencing factors of older adults’ cognitive function and depressive symptoms can guide targeted intervention measures to decelerate the decline of cognitive impairment and reduce depressive symptoms.

Most of the extant studies have used cross-sectional surveys to explore the correlation between depressive symptoms and cognitive impairment, with inconclusive results [10]. Some studies have associated the presence of depressive symptoms with cognitive decline or dementia [11, 12], some have found that the effect of depression on one cognitive ability (i.e., processing speed) has an additional effect on other cognitive abilities that interfere with everyday problem-solving [13,14], and some have found no correlation between these variables [15,16]. These inconsistent results have mostly been reported in studies that have concentrated on a single assessment (mostly at the baseline) of depressive symptoms and cognitive function decline. Considering that depressive symptoms tend to fluctuate over time, these studies have failed to fully capture the continuous impact of cognitive decline on these symptoms, thus limiting the understanding of the condition.

To the best of this study’s knowledge, only a few longitudinal studies have examined the relationship between cognitive function and depressive symptoms, and most have focused on the longitudinal effects of depressive symptoms on cognitive function [17,18]. For example, a longitudinal study with fewer than 400 participants purported an association between cognitive function and depressive symptoms in older adults [19] which limited the results’ universality. One study has suggested that there is no evidence of a relationship between cognitive function and depressive symptoms [20], while another has suggested a negative correlation between the two [21,22]. Moreover, some studies have examined the association between baseline depressive symptoms and incidental cognitive impairment [23–25] rather than a correlation among their trajectories. Others have evaluated cognitive function and depressive symptoms by repeatedly using cross-lag panel analysis, which has more application value in a two-way relationship; however, each result was exposed before the follow-up period, which did not exceed 3 years [19]. Understanding the changing trends of cognitive impairment and depressive symptoms could be improved by performing long-term follow-ups and repeated evaluations to further confirm their relationship. However, the longitudinal effects of cognitive function on depressive symptoms have not been fully investigated.

To further clarify whether cognitive function and other covariates impact depressive symptoms and to provide guidance for depression prevention measures, this study uses a latent growth curve model (LGCM) to analyze large longitudinal sample data for up to 7 years. An LGCM is an advanced analytical method that can create random intercepts and slopes to depict different trajectories over time [26]. It can also allow for the simultaneous estimation of individual and group variation and can be used to model aspects of change, describe a single individual trajectory, and capture individual differences in a trajectory over time [27].

To test whether cognitive function and other covariates are predictive of depressive symptoms development, this study uses an LGCM model based on three waves of the Chinese Longitudinal Healthy Longevity Study (CLHLS) data. The previous cognitive function- and depressive symptoms-related research on older adults has explored the following factors: exercise [28], sex [29], marital status [30], education [31,32], living conditions [33], and chronic diseases [34]. Therefore, this study examines whether these covariates confound the association, and their effect on the cognitive function and depressive symptoms trajectories.

## Methods

### Study Design and Participants

The study data were from the CLHLS, a large national survey of the Chinese population over the age of 65. Three waves of the CLHLS were used: 2011 (T1), 2014 (T2), and 2018 (T3). The samples were randomly selected from 22 of the 31 Chinese provinces (representing 85% of China’s total population) so as to provide a nationally representative study of older Chinese adults. The study conducted eight rounds of surveys in 1998, 2000, 2002, 2005, 2008, 2011, 2014, and 2018 [35]. During the surveys, face-to-face communication was conducted to collect information on the depressive symptoms and cognitive function of older adults. All investigators were subjected to unified training before starting the investigation to act as investigators when qualified. The CLHLS is an ongoing project, and a variety of mechanisms are in place to ensure the quality of the CLHLS data. Details regarding the sampling design and data quality mechanisms can be found elsewhere [36]. Informed consent was obtained from each respondent during the surveys [37]. After accounting for the dropouts and death of the older adults in the three waves, the samples comprised of 9765 participants in 2011, 7192 participants in 2014, and 15,874 participants in 2018. To merge the longitudinal data from the three waves, this study filtered for missing values, outliers, and the identity code (ID) was the only matching condition. This study gathered the data from the three waves to finally obtain a sample of 2454 participants who had participated in the CLHLS surveys (i.e., in 2011, 2014, and 2018), of which 1262 had provided valid questionnaires (i.e., they had participated in the three surveys and had answered the depressive symptoms and cognitive function scales completely). Missing values were imputed using multiple imputation. The included and excluded participants significantly differed in terms of sex, age, and chronic disease. The excluded participants (i.e., those who did not complete the depressive symptoms and cognitive function scales) were, on average, older (*p* < 0.001), female (*p* < 0.001), and had more chronic diseases (*p* < 0.001). Unsurprisingly, the included participants were younger and healthier when compared to the excluded participants, given the need to fulfill at least three waves of data collection spanning 7 years.

## Measures

### Depressive Symptoms

The CLHLS uses a five-item scale that assesses depressive symptoms in older adults; this scale has often been used to indicate depressive symptoms [2, 38–40]. Regarding the five items, the first two and last three items indicate positive and negative emotions, respectively. Positive items include “Do you see the bright side of things?” and “Do you feel the same level of happiness now as to when you were young?” Negative items include “Is anxiety a frequent occurrence for you?,” “Is it common for you to feel lonely and isolated?,” and “Is it true that the older you get, the less useful you seem to be?” To ensure measurement consistency, this study inversely assigned the two positive items as negative scores. The item responses were “Always,” “Often,” “Sometimes,” “Seldom,” and “Never,” and the scores ranged from 1–5, with a higher score indicating greater negativity. Therefore, this study calculated that the scores ranged from 5 to 25, with higher scores indicating more severe depressive symptoms. The Cronbach’s α coefficients of the three survey waves (2011, 2014, and 2018) were 0.66, 0.70, and 0.66, respectively, which were above the acceptable value of 0.6 [41]. The principle component analysis generated one factor with eigenvalues of ≥1, thus explaining 44.5% of the total variance [2].

### Cognitive Function

Cognitive function was assessed using the Chinese version of the Mini-Mental State Examination (CMMSE). The scale has good reliability and validity in the general population [42]. The sensitivity of the CMMSE is 95.9% and the specificity is 96.4%, which are both above 95% [43]; thus, the CMMSE is sensitive to changes over time in the older adult population. The CMMSE scale contains 24 questions on 5 dimensions: Orientation, language, recall, attention, and calculating ability. One point was allocated for a correct answer and zero points were allocated for incorrect answers, except for one question (“Please name as many kinds of food as possible in 1 min”; one point was given for each food named and seven points were given for those who named seven or more foods). The CMMSE scores ranged from 0 to 30, with higher scores denoting better cognitive function. The Cronbach’s α coefficients of the three survey waves (2011, 2014, and 2018) were 0.72, 0.72, and 0.81, respectively.

### Covariates

The covariates included personal physical factors (exercise), demographic factors (sex, marital status, and education), living conditions, and chronic diseases. All variables were measured during the first assessment (T1) and were regarded as covariates of the cognitive function and depressive symptoms intercept and slope factors, thus allowing for the examination of the effects of each covariate while controlling for all others. Males and females were coded as one and zero, respectively. Exercise was coded as one; no exercise was coded as zero. Regarding marital status, those who were divorced, widowed, and never married were coded as zero (i.e., no spouse); living with a spouse was coded as one (i.e., spouse). In the surveys, the question, “How many years did you go to school for?” was a continuous variable. Years of education was selected to reflect the education level of older adults, and was included in the model analysis. Regarding living conditions, those who lived alone or in nursing institutions were coded as zero (i.e., not living with family); living with family was coded as one. Chronic physical disease was regarded as a time-varying covariate and included hypertension, diabetes, heart disease, stroke, bronchitis, tuberculosis, cataracts, glaucoma, cancer, gastric or duodenal ulcer, Parkinson’s disease, bedsore, arthritis, and dementia. Chronic physical diseases were grouped as zero (i.e., no chronic disease), one (i.e., one chronic disease), and two (i.e., more than two chronic diseases).

### Data Analysis

This study used SPSS 25.0 and MPlus 8.1 software to build the LGCM. It defined the structure of two latent variables: the baseline level (intercept) and the change rate (slope). The mean intercept reflected the average of the individual baseline levels and the intercept variation reflected their heterogeneity. The synonymous slope mean reflected the overall change rate and the slope variation reflected the individual differences. This study evaluated the linear trajectory as the overall sample and discussed the potential influencing factors to analyze the individual differences between the baseline level of cognitive function, the potential factors of depressive symptoms, and the relationship between them, as well as the change rate over time [44]. This study estimated the linear variation between cognitive function and depressive symptoms to determine the degree of model fit. This study adopted the intercept and slope of cognitive function and depressive symptoms to evaluate the structural correlation between the two variables.

This study presented the participants’ characteristics, including cognitive function scores, depressive symptoms scores, and covariate distributions or scores, as descriptive statistics. The partial missing values of other covariates were interpolated by multiple interpolations [45]. Since the chi-square statistic is sensitive to sample size, this study assessed the model’s goodness of fit using the comparative fit index (CFI >0.90), Tucker-Lewis index (TLI >0.90), and root mean square error of approximation index (RMSEA <0.08) [46].

## Results

### Participants’ Characteristics

This study included 1,262 participants (661 males, 601 females). Overall, 41.5%, 29.3%, and 29.2% of the participants had never received an education, received an education for 5 years, and received an education for 6 years or more, respectively. Table 1 presents the other sample characteristics.

**TABLE 1:** Sample characteristics (Chinese Longitudinal Health Longevity Survey, China, 2011–2018).

Variables	n	%
Sex		
Male	661	52.4
Female	601	47.6
Exercise		
Yes	467	37.0
No	795	63.0
Marital status		
Having a spouse	853	67.6
No spouse	409	32.4
Education		
0	524	41.5
1–5	370	29.3
>5	368	29.2
living condition		
Living with the family	1085	86.0
Not living with the family	177	14.0

### Change Trend of Depressive Symptoms

The nonlinear curve model of depressive symptoms could not be fitted, so the linear model was chosen. Tables 2, 3 show the change trend of depressive symptoms in the time and unconditional linear models, respectively. The results demonstrate that depressive symptoms increases from the first to the third wave, and the index indicates a good model fit. The intercept (baseline) mean of depressive symptoms is 10.53 (*p* < 0.001), and its average slope is 0.06 (*p* < 0.001), indicating that older adults’ depressive symptoms show a linear upward trend first to the third wave. The variations in intercept and slope are 3.89 (*p* < 0.001) and 0.06 (*p* = 0.025), respectively, indicating significant individual differences in the baseline of depressive symptoms level and change rate of older adults.

**TABLE 2:** Mean and standard deviation by each variable (Chinese Longitudinal Health Longevity Survey, China, 2011–2018).

Variable	Time 1		Time 2		Time 3	
	M	SD	M	SD	M	SD
Depressive Symptoms	10.53	(3.21)	10.71	(3.26)	10.98	(3.16)
Cognitive function	27.93	(2.76)	27.81	(2.69)	27.03	(3.56)

**TABLE 3:** Results of each variable’s model suitability (Chinese Longitudinal Health Longevity Survey, China, 2011–2018).

Variable	2(df)	TLI	CFI	RMSEA	Intercept		Slope	
					Mean	Variance	Mean	Variance
Depressive Symptoms	0.02(1)	1.00	1.00	0.00	10.53	3.89	0.06	0.06
Cognitive function	11.64(1)	0.91	0.97	0.09	28.05	1.965	−0.13	0.03
Multivariate linearity LGM	64.61(24)	0.90	0.96	0.04				

### Change Trend of Cognitive Function

The nonlinear curve model of cognitive function could not be fitted, so the linear model was chosen. Tables 2, 3 show the change trend of cognitive function in the time and unconditional linear models, respectively. The results indicate that older adults’ cognitive function shows a declining trend during the 7-year observation period, and is more significant in the second and third waves. In the linear unconditional model, only the RMSEA value is slightly higher, and the other fitting indexes are good. The intercept (baseline) mean of cognitive function is 28.05 (*p* < 0.001). The average slope of cognitive function is −0.13 (*p* < 0.001), indicating that older adults’ cognitive function shows a linear downward trend during the 7-year observation period. A variation of 1.97 is shown between the intercept and slope, at (*p* < 0.001) and 0.03 (*p* > 0.05), respectively, indicating significant individual differences in the baseline cognitive function level and change rate of older adults. but the change in cognition speed is generally consistent.

### How do Cognitive Function and Covariates Longitudinally Affect Older Adults’ Depressive Symptoms?

This study used the LGCM to examine whether depressive symptoms changed over time as a result of cognitive function. All covariates were simultaneously entered into the model to allow for an examination of each covariate’s effect while controlling for all other covariates. In Table 3, all indices indicate that the final model fits well. The TLI, CFI, and RMSEA of the acceptable model are 0.90, 0.96, and 0.04, respectively. The final model indicates that the cognitive function intercept affects the depressive symptoms intercept (*β* = −0.42, *p* < 0.001) and slope (*β* = 0.42, *p* = 0.004). That is, the higher the baseline level of cognitive function, the lower the baseline level of depressive symptoms, and the faster the upward trend of depressive symptoms. There is a significant negative correlation between the cognitive function and depressive symptoms slope (*β* = −0.65, *p* = 0.002). Therefore, the faster the cognitive function decline, the faster the upward trend of depressive symptoms.

**FIGURE 1:**
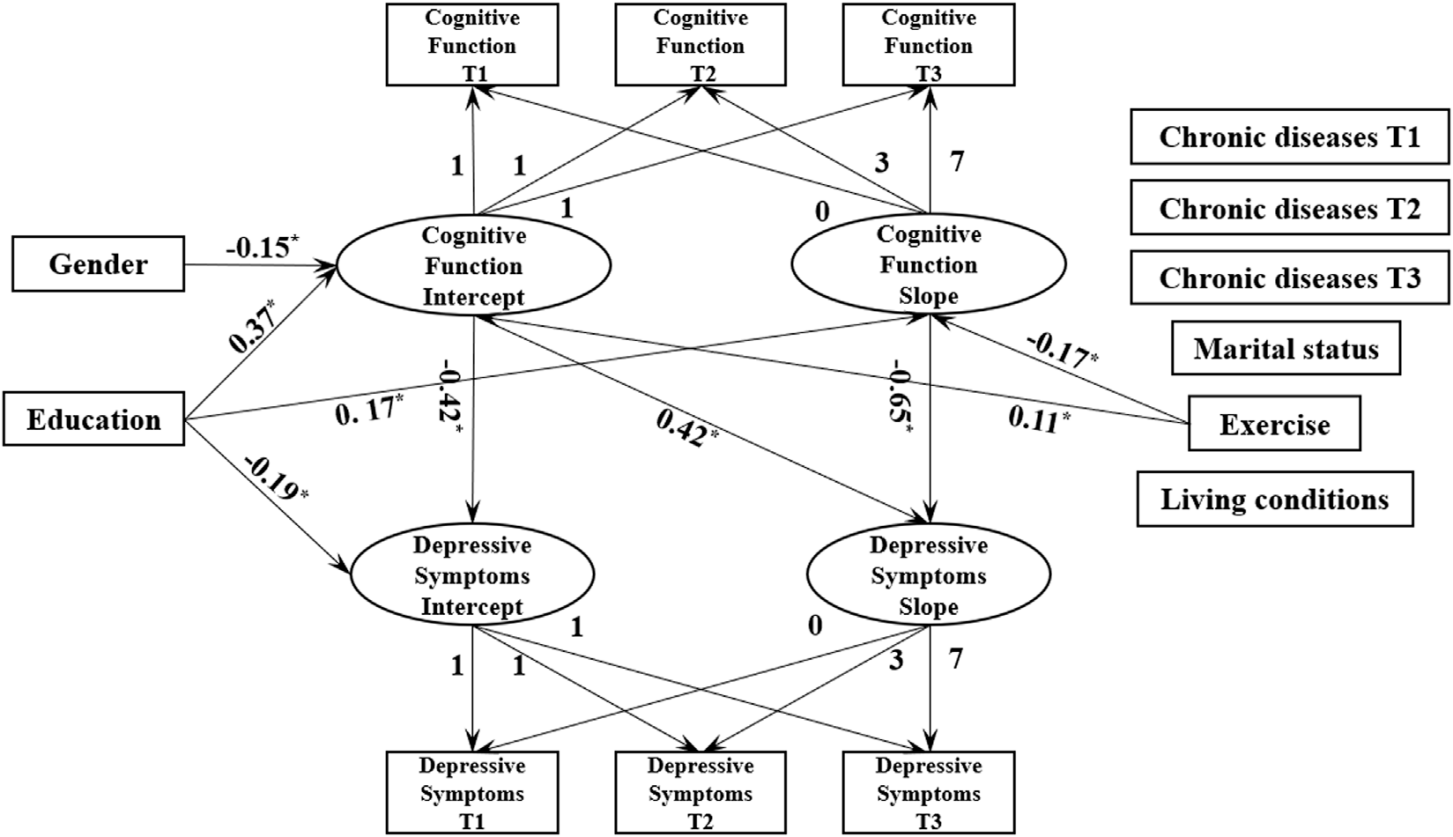
Multivariate linearity Latent Growth Curve Model (Chinese Longitudinal Health Longevity Survey, China, 2011–2018).

In Figure 1, the covariates show that sex has a significant impact on the intercept of the cognitive function trajectory (*β* = −0.15, *p* = 0.003), indicating that the baseline level of older adult female’s cognitive function is worse than that of older adult males. The depressive symptoms intercept and cognitive function slope are unaffected by sex. There is a positive correlation between education, the cognitive function intercept and slope (*β* = 0.37, *p* < 0.001; *β* = 0.17, *p* = 0.022), and the depressive symptoms intercept (*β* = −0.19, *p* = 0.003). Thus, the higher the education level, the better the cognitive function change rate, and the lower the baseline level of depressive symptoms. The cognitive function change rate is affected by education level. Thus, higher education levels are associated with faster cognitive function decline change rates. There is a positive correlation between exercise and the cognitive function intercept (*β* = 0.11, *p* < 0.021), but a negative correlation between exercise and the cognitive function slope (*β* = −0.17, *p* = 0.016). This suggests that older adults who exercise regularly have a higher baseline cognitive function level and a slower rate of cognitive function decline. Marital status, chronic diseases and living conditions do not affect the depressive symptoms and cognitive function trajectories.

## Discussion

This study used an LGCM to model aspects of change in the longitudinal trajectories of cognitive function and depressive symptoms, so as to describe individual trajectories and capture individual differences over time. The overall research framework included depressive symptoms, cognitive function, and the covariates to analyze the influencing characteristics of cognitive function and the covariates on depressive symptoms in older adults. Analyzing the relationship between cognitive function, depressive symptoms, and the covariates can remedy the lack of extant literature and provide important evidence. This study shows that older adults’ cognitive function can influence their development of depressive symptoms, to some extent, and the longitudinal results confirm the findings of previous cross-sectional analyses [47].

### Longitudinal Trajectories of Depressive Symptoms and Cognitive Function in Older Adults

This study reveals that older adults’ depressive symptoms increase linearly. When older adults enter advanced age, their organ function deteriorates, various chronic diseases follow, and economic burden increases. Hence, the emergence of disease expedites the reduction in older adults’ QOL and their feeling of missing the sense of novelty toward life [48]. The superposition of various problems also triggers an increase in the depression level in older adults. This study’s finding that older adults’ cognitive function decrease linearly is consistent with the previous studies that have suggested that the cognitive function of older adults will dramatically decline after the age of 60 [49]. A major risk factor for cognitive function decline is aging, which could be related to the decrease in brain function caused by advanced age and brain atrophy over time. A previous study has suggested that cognitive decline may be associated with the volume of thalamic atrophy [50]. Older adult patients with cognitive impairment and depressive symptoms should receive clinical attention. While focusing on their cognitive function, sufficient attention should also be paid to older adults’ mental health. Medical workers could provide older adults with health education related to cognitive function and mental health problems. Moreover, community nurses could formulate effective and dynamic assessment strategies to improve older adults’cognitive function and depressive symptoms, and encourage them to participate in cognitive function and psychological tests.

### Influential Relationship of Developmental Trajectory Between Older Adults’ Cognitive Function and Depressive Symptoms

This study reveals that the baseline level of cognitive function in older adults can significantly predict the baseline value and change rate of depressive symptoms. The higher the baseline level of cognitive function, the lower the baseline level of depressive symptoms, but the faster the growth of depressive symptoms in the process of its subsequent development. Moreover, the faster the cognitive function decline, the faster the upward trend of depressive symptoms. Researchers have examined the association between cumulative depressive symptoms and cognitive decline in several cohort studies, with inconsistent conclusions. For example, some studies have reported a link between depressive symptoms and cognitive function [47, 51, 52], while others have found no association [20, 53], or an association in sub-groups only [54]. Some cross-sectional studies have shown that older adults’ cognitive function negatively affected their depressive symptoms [54, 55]; moreover, patients with depression have decreased working memory and attention, and the severity is negatively correlated with depressive symptoms. The awareness of cognitive decline may cause depression as a psychological reaction to the loss of cognitive function [56]. Therefore, people with cognitive impairment have a higher incidence of depression, and after their cognitive function is impaired, depression will occur. Normal cognitive function can help them buffer the painful experience caused by depressive symptoms. Therefore, based on the characteristics of older adults in the community, a comprehensive intervention program should be constructed that includes cognitive impairment and depression to promote the development of healthy aging. Moreover, nursing professionals and family caregivers should help older adults to retain a favorable level of cognitive function by cultivating cognitive training, so as to reduce their depressive symptoms level.

### Related Factors Influencing the Depressive Symptoms and Cognitive Function Trajectories in Older Adults

Epidemiological studies have suggested that long-term exposure to complex functional and leisure environments or social activities can enhance cognitive function [57]. In traditional Chinese society and culture, males are exposed to such activities or environments for longer periods, which plays an affirmative role in their cognitive function reserve and prevents neurological decline [58].

The current study reveals that the higher the older adults’ education level, the higher their baseline cognitive function, and the lower their baseline depressive symptoms. A previous study has suggested that uneducated older adults are three to four times more likely to develop cognitive impairment than those who have received an education [59]. The current study further supports the idea that a few years of schooling can have a protective influence on cognitive dysfunction, since older adults with more years of education have higher cognitive function.

This study demonstrates that exercise has a positive effect on older adults’ baseline cognitive function, while cognitive function decline is slower in those who exercise. Similar findings have been found in other studies [18,60]. According to a randomized controlled trial of middle-aged and older adults, exercise can have a beneficial effect on specific aspects of cognitive function [18,61]; the current study’s conclusions are consistent with this finding. Hence, exercise may play an important role in promoting older adults’ cognitive function as it can indirectly reduce cognitive function decline and improve cognitive function.

### Implications for Practice

This study shows that depression and cognitive impairment are persistent problems among older adults. Nevertheless, only 2.4% of older adults are diagnosed as having depression [62]. Therefore, there is an urgent need to vigorously build a team of mental health nursing personnel and improve the professionalism of nursing services to cope with the increasing demand for mental health services for older adults.

This study suggests that nursing professionals and family caregivers should pay special attention to the psychological conditions of older adults with rapidly declining cognitive function because the increase of depressive symptoms can occur during this critical period. Early detection and intervention of predictive indicators based on cognitive function could be effective in preventing the increase of depression levels, delaying the progress of depression, and reducing its prevalence and related public health burden. Moreover, the community could regularly guide older adults in community exercise activities, so as to sequentially slow down their cognitive decline and maximize the benefits of physical and mental health.

### Limitations and Future Perspectives

This study has the following limitations. First, the measurement of depressive symptoms cannot completely represent the clinical diagnosis and assessment of depression. Second, the use of the CMMSE to characterize cognitive function may be associated with ceiling effects and inadequate sensitivity in detecting subtle cognitive impairment. The future studies would benefit from the use of more sensitive scales to identify depressive symptoms and cognitive function. Third, this study excluded people who were older and who had more chronic diseases, so it is necessary to further explore the longitudinal association between depressive symptoms and cognitive function in this group in the future.

### Conclusion

This study uses an LGCM to explore whether changes in depressive symptoms over time are associated with cognitive function. The findings may have clinical implications for healthcare providers involved with depressive symptoms prevention or care. Notably, the decline rate of cognitive function in older adults can effectively predict the growth rate of depressive symptoms. Education and exercise can effectively ameliorate older adults’depressive symptoms. The findings can help nursing staff and health managers to address this situation., so as to reinforce the prevention of depressive symptoms in older adults and take targeted measures to reduce their risk of depression by promoting healthy exercise.

## Supplementary Material

Supplementary Table S1 Standardised coefficients for covariates in the multivariate linearity latent growth curve model (Chinese Longitudinal Health Longevity Survey, China, 2011–2018).
