# Corrigendum: Longitudinal Association Between Depressive Symptoms and Cognitive Function Among Older Adults: A Parallel Latent Growth Curve Modeling Approach

**DOI:** 10.3389/ijph.2022.1605525

**Published:** 2022-12-01

**Authors:** Zihan Gao, Cuiping Liu, Li Yang, Xinyi Mei, Xiao Wei, Jinke Kuang, Kexin Zhou, Mengfan Xu

**Affiliations:** ^1^ School of Nursing, Qingdao University, Qingdao, China; ^2^ School of Nursing, Wuhan University of Science and Technology, Wuhan, China

**Keywords:** longitudinal study, cognitive function, depressive symptoms, latent growth curve model, old adults

A correction has been made to the section **Measures**, sub-section *Depressive symptoms*. An incorrect range of scores was included in the original publication. The erroneous text was:

“The item responses were “Always,” “Often,” “Sometimes,” “Seldom,” and “Never,” and the scores ranged from 1–5, with a higher score indicating greater negativity. Therefore, this study calculated that the scores ranged from 0 to 25, with higher scores indicating more severe depressive symptoms.”

The erroneous text has been corrected. The corrected sentence appears below: 

“The item responses were “Always,” “Often,” “Sometimes,” “Seldom,” and “Never,” and the scores ranged from 1–5, with a higher score indicating greater negativity. Therefore, this study calculated that the scores ranged from 5 to 25, with higher scores indicating more severe depressive symptoms.”

The authors apologize for this error and state that this does not change the scientific conclusions of the article in any way. The original article has been updated.

