# Supplementary material for: Longitudinal Association Between Depressive Symptoms and Cognitive Function Among Older Adults: A Latent Growth Curve Modeling Approach
Source: Int J Public Health. 2022 Sep 23;67:1605124. doi: 10.3389/ijph.2022.1605124 (PMC9537360; doi:10.3389/ijph.2022.1605124)
Supplement: Supplementary file 1 [file Table1.DOCX]

Table 1. Standardised coefficients for covariates in the multivariate linearity latent growth curve model.

| Correlations | β | *P* |
| --- | --- | --- |
| CF Slope→DS Slope | -0.65* | 0.002 |
| CF Intercept →DS Slope | 0.42* | 0.004 |
| CF Slope →DS Intercept | 0.22 | 0.057 |
| CF Intercept →DS Intercept | -0.42* | <0.001 |
| Gender →CF Intercept | -0.15* | 0.003 |
| Education →CF Intercept | 0.37* | <0.001 |
| Marital status→CF Intercept | 0.09 | 0.115 |
| Exercise→CF Intercept | 0.11* | 0.021 |
| Living conditions→CF Intercept | -0.01 | 0.906 |
| Numbers of chronic diseases 1→CF Intercept | -0.00 | 0.932 |
| Numbers of chronic diseases 2→CF Intercept | -0.02 | 0.725 |
| Numbers of chronic diseases 3→CF Intercept | 0.03 | 0.560 |
| Gender →CF Slope | -0.06 | 0.419 |
| Education →CF Slope | 0.17* | 0.022 |
| Marital status→CF Slope | 0.12 | 0.159 |
| Exercise→CF Slope | -0.17* | 0.016 |
| Living conditions→CF Slope | 0.01 | 0.879 |
| Numbers of chronic diseases 1→CF Slope | 0.08 | 0.248 |
| Numbers of chronic diseases 2→CF Slope | -0.10 | 0.160 |
| Numbers of chronic diseases 3→CF Slope | -0.09 | 0.182 |
| Gender →DS Intercept | -0.04 | 0.495 |
| Education →DS Intercept | -0.19* | 0.003 |
| Marital status→DS Intercept | -0.05 | 0.395 |
| Exercise→DS Intercept | -0.05 | 0.342 |
| Living conditions→DS Intercept | -0.01 | 0.908 |
| Numbers of chronic diseases 1→DS Intercept | -0.03 | 0.575 |
| Numbers of chronic diseases 2→DS Intercept | 0.10 | 0.063 |
| Numbers of chronic diseases 3→DS Intercept | 0.04 | 0.400 |
| Gender →DS Slope | 0.01 | 0.881 |
| Education →DS Slope | 0.06 | 0.584 |
| Marital status→DS Slope | -0.02 | 0.832 |
| Exercise→DS Slope | -0.07 | 0.456 |
| Living conditions→DS Slope | 0.07 | 0.473 |
| Numbers of chronic diseases 1→DS Slope | 0.11 | 0.197 |
| Numbers of chronic diseases 2→DS Slope | -0.09 | 0.275 |
| Numbers of chronic diseases 3→DS Slope | 0.05 | 0.560 |

*Notes.* Abbreviations: DS: depressive symptoms; CF; cognitive function;

1: year 2011; 2: year 2014; 3: year 2018.

*p < 0.05.
